# Treatment completion for latent tuberculosis infection: a retrospective cohort study comparing 9 months of isoniazid, 4 months of rifampin and 3 months of isoniazid and rifapentine

**DOI:** 10.1186/s12879-017-2245-8

**Published:** 2017-02-14

**Authors:** Adelaide H. McClintock, McKenna Eastment, Christy M. McKinney, Caroline L. Pitney, Masahiro Narita, David R. Park, Shireesha Dhanireddy, Alexandra Molnar

**Affiliations:** 1 Division of General Internal Medicine, Department of Medicine, University of Washington School of Medicine, Box 354765, 4245 Roosevelt Way NE, Seattle, WA 98105 USA; 2 Division of Allergy & Infectious Diseases, Department of Medicine, University of Washington School of Medicine, Seattle, USA; 3 Pharmacy Department, Harborview Medical Center, Seattle, USA; 4 Division of Pulmonary & Critical Care Medicine, Department of Medicine, University of Washington School of Medicine, Seattle, USA; 5 Tuberculosis Control Program, Public Health - Seattle & King County, Seattle, USA

**Keywords:** Latent Tuberculosis Infection, Rifampin, Isoniazid, Rifapentine, Treatment adherence

## Abstract

**Background:**

The U.S. Centers for Disease Control and Prevention (CDC) recommended a new regimen for treatment of latent tuberculosis (three months of weekly isoniazid and rifapentine) in late 2011. While completion rates of this regimen were reported to be higher than nine months of isoniazid, little is known about the completion rates of three months of isoniazid and rifapentine compared to nine months of isoniazid or four months of rifampin in actual use scenarios.

**Methods:**

We conducted a retrospective cohort study comparing treatment completion for latent tuberculosis (TB) infection in patients treated with nine months of isoniazid, three months of isoniazid and rifapentine or four months of rifampin in outpatient clinics and a public health TB clinic in Seattle, Washington. The primary outcome of treatment completion was defined as 270 doses of isoniazid within 12 months, 120 doses of rifampin within six months and 12 doses of isoniazid and rifapentine within four months.

**Results:**

Three hundred ninety-three patients were included in the study. Patients were equally likely to complete three months of weekly isoniazid and rifapentine or four months of rifampin (85% completion rate of both regimens), as compared to 52% in the nine months of isoniazid group (*p* < 0.001). These associations remained statistically significant even after adjusting for clinic location and type of monitoring. Monitoring type (weekly versus monthly versus less often than monthly) had less impact on treatment completion than the type of treatment offered.

**Conclusions:**

Patients were equally as likely to complete the three months of isoniazid and rifapentine as four months of rifampin. Four months of rifampin is similar in efficacy compared to placebo as isoniazid and rifapentine but does not require directly observed therapy (DOT), and is less expensive compared to combination therapy with isoniazid and rifapentine, and thus can be the optimal treatment regimen to achieve the maximal efficacy in a community setting.

## Background

Treating latent TB infection (LTBI) in high-risk individuals is one of the eight Millennium Development goals of the United Nations [1]. In the United States alone, 9,421 cases of active TB were reported in 2014 [2], and it is estimated that more than 13 million people have latent TB infection [3, 4]. Aggressive treatment of LTBI provides significant cost-savings and quality-adjusted life years saved because it dramatically reduces the risk of progression to active TB [5]. Most of the cases in the United States occur in foreign born individuals [6]. In fact, TB in foreign born individuals is on the rise in the US [6]. Primary care and Public Health clinics are often the first contact newly arrived patients have with the health care system for screening for TB infection.

With the availability of novel treatment regimens for LTBI, medical providers now face the challenge of choosing which regimen is best suited for an individual patient. Historically, the regimen of choice for LTBI was nine-months of daily isoniazid [7]. Nine months of isoniazid reduces the rate of progression to active TB by up to 39-86%, but only if the patient is fully adherent [7, 8]. In most actual use studies, isoniazid completion rates are only 31-59% [9–11] making it a less effective treatment choice in non-research settings [7]. Additionally, use is limited by the concerns for side effects such as hepatotoxicity and neuropathy [7].

In 2011, the CDC recommended a three-month directly observed, weekly dosed therapy of isoniazid and rifapentine as an “equal alternative” to the nine month isoniazid regimen [12] in healthy individuals with LTBI. Based on three randomized controlled trials [13–15], isoniazid and rifapentine with directly observed therapy (DOT) is considered to be non- inferior to standard self-administered isoniazid for nine months in preventing progression to active TB, and more likely to be completed than isoniazid [12, 15]. Additionally, there is an alternative LTBI regimen of 3 months of daily rifampin and isoniazid. This regimen is used in parts of Europe and also carries the benefits of short duration and low cost. [16] However, the regimen is not currently on the list of CDC recommended regimens, and is not commonly used in the US at this time, and so was not included in this study.

There has been significant excitement about three months of isoniazid and rifapentine as an expedited treatment option, but many features of this treatment make it less than ideal for certain patients and clinical settings. While the isoniazid and rifapentine regimen offers the promise of higher completion rates, likely due to less cumbersome dosing and shorter duration, this therapy is also significantly more expensive likely driven by the recommended use of DOT [17–19], which may be inconvenient for some patients, and requires a large resource commitment for the clinics that administer it.

Daily rifampin for four months has long been seen as an alternative regimen [7] for people who cannot tolerate isoniazid for nine months, or who were exposed to INH-resistant TB. Prior studies have demonstrated higher completion rates with shorter rifampin-based regimens compared to isoniazid [11, 20, 21]. Additionally, in a recent review of LTBI treatment, rifampin was again introduced as a potential regimen with increased completion rates compared to nine months of isoniazid, though no direct comparisons were made between rifampin and isoniazid-rifapentine [22]. With the introduction of isoniazid plus rifapentine and rifampin only regimens, isoniazid only therapy use appears to have declined making a study of completion rates among these regimens particularly useful.

### Objective

To date, there have been no studies directly comparing completion rates of isoniazid only, rifampin only, and isoniazid and rifapentine regimens. Data on “actual use” in the outpatient setting would be helpful to health care providers to guide choice of therapy for individual patients. The aim of this study is to compare the rates of completion of the three LTBI therapies across heterogeneous populations in various outpatient settings, including primary care clinics, subspecialty clinics, and a public health TB clinic.

## Methods

We conducted a retrospective cohort study of all patients who initiated treatment for LTBI with nine months of isoniazid, rifampin only, or isoniazid and rifapentine dispensed at a hospital affiliated pharmacy or TB clinic during 2009 or between July 1, 2013 and June 30, 2014. Five hospital-affiliated clinics in Seattle, Washington participated in this study: four outpatient clinics affiliated with an urban academic tertiary care medical center, and one county TB clinic. The outpatient clinics include a hospital employee health clinic, a primary care clinic focused on care of immigrants and refugees, a primary care clinic focused on the care of homeless individuals, and a sub-specialty infectious diseases clinic. Given that this was a retrospective chart review and patient identifiers were removed before entry into the secure database, subject consent to participate was waived by the Institutional Review Board (IRB) per standard practices at our institution. The IRB at the University of Washington Human Subjects Division approved this study (#43613).

Patients were identified for inclusion in the study if they were 18 years old or older. For patients treated in the hospital-affiliated outpatient clinics, pharmacy records were used to identify patients who were given a prescription for isoniazid without ethambutol or rifampin, rifampin alone, or isoniazid with rifapentine. For isoniazid monotherapy patients, records were queried for those who initiated treatment in 2009, well before the nation-wide isoniazid shortage and 9 months of isoniazid was standard of care. Records were queried again for those who initiated treatment between July 1, 2013 and June 30, 2014 for all three regimens. Medical records for the TB clinic were held separately and LTBI cases treated with the regimens of interest were identified in a TB Control Program database. Diagnosis of LTBI was made by either tuberculin skin test (TST) or interferon gamma release assay (IGRA) in combination with chest x-ray. Screening for active and latent TB was standard practice for patients in the international clinic, homeless clinic and employee health due to the high risk of exposure in those populations. The infectious diseases clinic treated patients planning to undergo treatment of another disease process with immunosuppressive therapies. Patients from the Public Health Clinic, were screened as contacts of active cases being treated by Public Health. The sample size we had was determined by the number of patients seen in our clinics during our study period and so we did not calculate power. The 95% confidence intervals we present in Table 2 provide the best range of plausible estimates. Only one person was excluded from our chart review because it was determined upon further chart review that the participant had already completed therapy for LTBI prior to our study time frame. Patients with HIV were not included in this study, as this medical center has a separate HIV clinic which sees HIV patients, and because of the potential drug-drug interactions between antiretroviral therapy and rifamycin-based regimens.

Patients were evaluated for LTBI treatment and monitored for adverse effects during follow up visits. When evaluating for side effects, specific side effects such as liver function test (LFT) abnormalities, lab abnormalities, and other known or common medication side effects were marked as present or absent, whereas patient side effects were classified as “other” if the patient reported a subjective side effect which was not a known side effect of the medication regimens (for example, a report of dizziness after taking medications was classified as “other.”) Choice of treatment regimen, frequency of follow-up visits, and frequency of laboratory monitoring were selected by the treating physician at his or her discretion based on patients’ other comorbidities, medications, length of therapy and cost. Patients on the nine-month isoniazid and four-month rifampin daily regimens were typically seen monthly in the public health clinics, and monthly or less often in the outpatient clinics. Patients monitored less than monthly were monitored this way because of poor attendance in clinic. When present in clinic, regular monitoring was completed as it would have been in a monthly monitoring scenario. Patients on isoniazid and rifapentine were seen weekly for DOT by either a nurse or an outreach worker. A small number of the patients on isoniazid and rifapentine received weekly phone calls or met virtually with an outreach worker via webcam, rather than in-person contact. The public health TB clinic used a standard set of questions to check for specific side effects, whereas in all other clinics, patients were asked a targeted review of systems at the provider’s discretion to assess for side effects to the regimens. Interpreters were used for non-English-speaking patients.

The primary outcome was treatment completion. This outcome was determined based on pharmacy records of prescription fulfillment. Patients were considered to have completed therapy if they filled prescriptions consecutively to correspond to 270 doses of isoniazid within 12 months [7], 120 doses of rifampin in a 6 month period [7] or 12 doses of isoniazid and rifapentine in a 4 month period [12].

We calculated counts, proportions, and means for demographic, treatment, and health characteristics for all participants combined and by treatment type. We estimated counts and row proportions for treatment, clinic, and monitoring type. We hypothesized these three factors would be associated with completion of treatment. We used Poisson regression models with a log link and robust error variance to estimate unadjusted and adjusted relative risks (RR) along with 95% confidence intervals (CI) and *p*-values in lieu of logistic regression since our outcome was common. We identified potential confounders *a priori* based on their relationship between the hypothesized factor and completion of treatment. Confounders evaluated were specific to each exposure of interest and included demographic (e.g. age, type of insurance) and treatment characteristics (e.g. clinic, treatment regimen, monitoring type) and were modeled as categorized in Table 1. Potential confounders that altered estimates, generally by more than 10%, were retained in the adjusted models. Specifically, when determining the association between completion of therapy and treatment regimen, we adjusted for frequency of monitoring and when examining the association between completion of therapy and type of monitoring, we adjusted for type of monitoring. Because type of monitoring and treatment regimen may be related, we conducted sensitivity analyses for these associations with and without adjustment for type of monitoring and treatment regimen, respectively. Additionally, we conducted further sensitivity analyses post-hoc after noting differences across treatment regimen in homelessness and alcohol use. All analyses were conducted using Stata 13.1 [23]. The University of Washington Human Subjects Division reviewed and approved this research.

**Table 1:** Characteristics among all participants and stratified on treatment

	All Participants (*N* = 393)	Isoniazid + Rifapentine, 2013 only (*N* = 87)	Rifampin only, 2013 only (*N* = 82)	Isoniazid only, 2009 and 2013 (*N* = 224)^a^	*p*-value
	*n* (%)^b^	*n* (%)^b^	*n* (%)^b^	*n* (%)^b^	
Gender^c^					0.93
Female	175 (44.5)	38 (43.7)	38 (46.3)	99 (44.2)	
Age (years)^c^	43.6 (15.0)	43.2 (15.4)	43.4 (13.1)	43.8 (15.5)	0.94
Insurance^d^					0.19
Private	59 (18.0)	10 (15.2)	13 (22.0)	36 (17.8)	
Government sponsored	76 (23.2)	16 (24.2)	17 (28.8)	43 (21.3)	
Charity care	163 (49.9)	34 (51.5)	29 (49.2)	100 (49.5)	
Other	29 (8.9)	6 (9.1)	0 (0.0)	23 (11.4)	
Race/Ethnicity^c^					<0.001
Caucasian	30 (7.9)	8 (9.9)	3 (3.7)	19 (8.8)	
African or African American	94 (24.9)	18 (22.2)	20 (24.4)	56 (26.1)	
Asian	161 (42.6)	19 (23.5)	40 (48.8)	102 (47.4)	
Mexican or Other Hispanic	49 (13.0)	11 (13.6)	10 (12.2)	28 (13.0)	
Pacific Islander	32 (8.5)	21 (25.9)	6 (7.3)	5 (2.3)	
Other	12 (3.2)	4 (4.9)	3 (3.7)	5 (2.3)	
Foreign Born^c^	341 (90.7)	65 (79.3)	79 (98.8)	197 (92.1)	<0.001
English Speaking^c^	203 (52.5)	60 (70.6)	38 (46.3)	105 (47.7)	0.001
Homeless/marginally house^e^	45 (12.3)	24 (31.2)	2 (2.5)	19 (9.1)	<0.001
Number of other medical problems^c^					0.01
0	178 (45.3)	38 (43.7)	51 (62.2)	89 (39.7)	
1-2	119 (30.3)	29 (33.3)	19 (23.2)	71 (31.7)	
3+	96 (24.4)	20 (23.0)	12 (14.6)	64 (28.6)	
Number of medications^c^					<0.001
0-1	139 (35.4)	0 (0.0)	56 (68.3)	83 (37.1)	
2-3	138 (35.1)	57 (65.5)	12 (14.6)	69 (30.8)	
3+	116 (29.5)	30 (34.5)	14 (17.1)	72 (32.1)	
Any tobacco use at start of therapy^c^	49 (12.5)	19 (21.8)	11 (13.4)	19 (8.5)	0.01
Any alcohol at start of therapy^c^	52 (13.2)	21 (24.1)	10 (12.2)	21 (9.4)	0.002
Clinic^c^					<0.001
Infectious Diseases	17 (4.3)	8 (9.2)	0 (0.0)	9 (4.0)	
Public Health	263 (66.9)	69 (79.3)	67 (81.7)	127 (56.7)	
International Clinic	68 (17.3)	0 (0.0)	15 (18.3)	53 (23.7)	
Homeless Clinic	18 (4.6)	5 (5.8)	0 (0.0)	9 (4.0)	
Employee Health	27 (6.9)	5 (5.8)	0 (0.0)	22 (9.8)	
Type of Monitoring^c^					<0.001
Weekly DOT/Phone calls	87 (22.2)	84 (96.6)	1 (1.2)	2 (0.9)	
Monthly visit	139 (35.5)	2 (2.3)	66 (80.5)	71 (31.8)	
Less often	166 (42.4)	1 (1.2)	15 (18.3)	150 (67.3)	

^a^ 205/224 were treated in 2009 and 19/224 were treated in 2013. In 2009, 7/9 patients were treated in the Infectious Diseases Clinic, 123/127 at the Public Health clinic, 48/53 in International Clinic, 10/13 in the Homeless Clinic, and 17/22 in Employee Health

^b^ All estimates are percents except for age which is a mean (standard deviation)

^c^ Less than 5% of data missing; ^d^ 16.8% of data are missing; ^e^ 7.1% of data are missing

## Results

A total of 393 participants were included in the study: 87 patients received three months of isoniazid and rifapentine, 82 received four months of rifampin and 224 received nine months of isoniazid (Table 1). There were more men represented in this study than women (55.5% compared to 44.5%). The average age of study participants was 43.6 years. Foreign-born and non-English speaking participants were less likely to receive isoniazid and rifapentine. Treatment regimens differed by clinic and type of monitoring.

Patients treated with four months of rifampin were as likely to complete therapy as those on three months of isoniazid and rifapentine. Patients on nine months of isoniazid were less likely to complete treatment than patients on four months of rifampin or three months of isoniazid and rifapentine with adjusted relative risk of 0.66 (95% CI 0.56, 0.79) and 0.59 (95% CI 0.36, 0.93), respectively (Table 2). These associations remained statistically significant and in the direction anticipated after adjusting for clinic location and type of monitoring. In our sensitivity analyses, this association between treatment regimen and completion was similar with and without adjustment for type of monitoring.

**Table 2:** Factors associated with completion of therapy

	Not Completed		Completed		Unadjusted			Adjusted		
	*n*	percent	*n*	percent	RR	95% CI	*p*-value	RR	95% CI	*p*-value
Type of Therapy^a,b^										
Isoniziad + Rifpaentine	13	14.9	74	85.1	Reference			Reference		
Rifampin only	12	14.6	70	85.4	1.00	0.88, 1.14	0.96	0.88	0.55, 1.38	0.57
Isoniazid only	107	48.2	115	51.8	0.61	0.52, 0.71	<0.001	0.58	0.36, 0.93	0.02
Isoniazid only (vs rifampin only)					0.61	0.52, 0.71	<0.001	0.66	0.56, 0.79	<0.001
Clinic^b,c^										
Public Health	59	22.5	203	77.5	Reference			Reference		
Employee Health	20	74.1	7	25.9	0.33	0.18, 0.64	<0.001	0.37	0.20, 0.69	<0.01
International	37	54.4	31	45.6	0.59	0.45, 0.77	<0.001	0.64	0.47, 0.85	<0.01
Homeless	9	52.9	8	47.1	0.61	0.37, 1.01	0.06	0.65	0.38, 1.09	0.10
Infectious Diseases	7	41.2	10	58.8	0.76	0.51, 1.14	0.18	0.74	0.48, 1.12	0.16
Type of monitoring^a,c^										
Weekly DOT/phone calls	13	15.1	73	84.9	Reference			Reference		
Monthly clinic visits	38	27.3	101	72.7	0.86	0.75, 0.98	0.03	1.12	0.71, 1.76	0.62
Less often	81	48.8	85	51.2	0.60	0.51, 0.72	<0.001	1.21	0.75, 1.96	0.43
Side effects^c^	37	28.2	94	71.8	1.13	0.98, 1.30	0.09	1.03	0.90, 1.18	0.66
Number of other medical problems^a,d^										
0	47	26.6	130	73.5	Reference			Reference		
1-2	42	35.3	77	64.7	0.88	0.75, 1.03	0.12	1.00	0.84, 1.19	1.00
3+	43	45.3	52	54.7	0.75	0.61, 0.91	0.01	1.08	0.88, 1.33	0.47

*Abbreviations: RR* Relative Risk, *95% CI* 95% Confidence Interval

^a^ Adjusted for clinic type

^b^ Adjusted for type of monitoring

^c^ Adjusted for treatment

^d^ Adjusted for insurance

Patients treated in the employee or refugee clinics were less likely than patients in the public health TB clinic to complete therapy with adjusted RR of 0.37 (95% CI 0.20, 0.69) and 0.64 (95% CI 0.47, 0.85) respectively (all *p*-values ≤0.01). Patients treated in the homeless clinic or infectious diseases clinic appeared to be less likely to complete treatment than patients in the public health TB clinic, although the differences were not statistically significant with adjusted RR of 0.65 (95% CI 0.38, 1.09) and RR of 0.74 (95% CI 0.48, 1.12), respectively (Table 2).

In unadjusted analyses, patients with monthly or less frequent monitoring were less likely to complete therapy than those with weekly DOT with RR of 0.86 (95% CI 0.75, 0.98) and RR of 0.60 (95% CI 0.51, 0.72). However, these associations were no longer apparent after adjusting for clinic location and treatment type with RR of 1.12 (95% CI 0.721, 1.76) and RR of 1.21 (95% CI 0.48, 1.12), respectively (Table 2). In sensitivity analyses that examined this association between frequency of monitoring and treatment completion without adjusting for treatment regimen, there is a significant association with RR of monthly monitoring of 0.82 (95% CI 0.72, 0.94) and less frequent monitoring of 0.73 (95% CI 0.61, 0.89) compared to weekly DOT. This difference in association with and without adjustment for treatment regimen is likely related to the strong association between treatment regimen and monitoring type.

We evaluated race/ethnicity, foreign born status and English-speaking for all adjusted associations presented in Table 2. None of these variables altered our associations or inference (*p*-values, confidence intervals) in any material way (e.g. there was <10% change in our estimates and no change in our *p*-values being above or below 0.05). We did not adjust for these factors in our analysis since they did not confound our associations. Additionally, we compared our reported results for all adjusted associations in Table 2 with models that also adjusted for homelessness and alcohol use. We observed no material difference in the associations (all were in the same direction and statistical significance did not change).

There was no difference in reporting of any side effects in those who completed versus those who did not complete therapy with RR of 1.03 (95% CI 0.90, 1.18) (Table 2). We hypothesized *a priori* that those patients with three or more medical problems would complete therapy less often. However, after adjusting for the clinic type and insurance, there was no significant difference in completion rates between patients with more medical problems compared to patients with fewer medical problems with RR of 1.08 (95% CI 0.88, 1.33) (Table 2).

### Key Findings

This retrospective cohort study demonstrated comparable completion rates between the novel three months of isoniazid and rifapentine and the less often used four months of rifampin. Both regimens had superior rates of completion compared to nine months of isoniazid. Currently, four months of rifampin is considered a secondary alternative, rather than a comparable choice with nine months of isoniazid or 12 weeks of rifapentine with isoniazid, which are the current standard. Given the lower cost to patients and health care settings of four months of rifampin compared to three months of isoniazid and rifapentine, we suggest that providers consider four months of rifampin as an alternative first-line option for LTBI treatment among the appropriate populations. Use of rifampin would offer significant cost savings to patients and health care systems in the United States and abroad. Additionally, improved rates of treatment completion would help reduce incidence of active cases in the United States and around the world.

## Discussion

With the publication in 2011 of non-inferiority of three months of isoniazid and rifapentine [15] to nine months of isoniazid, many have brought this treatment into the armamentarium against LTBI. Subsequent statistical modeling exercises suggest that three months of isoniazid and rifapentine is more effective and saves money compared to nine months of isoniazid because of higher rates of treatment completion and subsequent prevention of activation of TB [19, 24]. With this retrospective cohort study, we confirmed prior findings that three months of isoniazid and rifapentine is associated with higher completion rates relative to nine months of isoniazid. Additionally, these findings were true across a variety of patient populations and outpatient practice settings, reinforcing that three months of isoniazid and rifapentine is an excellent treatment option for patients who are not likely to adhere to nine months of isoniazid.

New and exciting treatments may not always be the best choice [25]. Rifamycin-based regimens are known to have low toxicity [8], however, all rifamycin based regimens share similar side effect profiles, including discoloration of bodily fluids (a use-limiting effect for some patients), and interactions with many common medications, including anticoagulants, HIV medications, and contraception.

The results of this study have important practical implications for providers, patients, and health care systems. The two regimens likely have similar side effect profiles [15, 26], but four months of rifampin does not require DOT and is less expensive than three months of isoniazid and rifapentine. Both four months of rifampin [8] and three months of isoniazid plus rifapentine [15] are also highly effective against latent TB, with similar efficacy compared to placebo or isoniazid monotherapy, respectively. While rifampin-only regimens are currently considered an alternative regimen, our findings suggest that rifampin-only regimens should be considered not just as an alternative to nine months of isoniazid, but as a comparable first-line regimen to three months of isoniazid and rifapentine and perhaps even superior to nine months of isoniazid given much better completion rates.

Our data show that the type of treatment offered was a strong predictor of treatment completion whereas monitoring type was not. This is contrary to our *a priori* hypothesis in which we anticipated DOT regimens would be superior due to the nature of DOT. No significant difference was found in completion rates between DOT and the rifampin-based self-administered regimen. In other words, non-adherence to the recommended follow-up was not associated with treatment non-completion. Our data represent real-life scenarios that are often missing from research settings, which can make research findings challenging to apply to the imperfect clinical setting. These findings suggest that self-administered four months of rifampin may be able achieve high compliance rates similar to the rates of three months of isoniazid and rifapentine, without the costs of DOT to both patients and health care systems. These findings underscore the utility of four months of rifampin for providers who do not have the resources available for DOT in the outpatient setting.

We also found significantly higher completion rates at the public health TB clinic compared to primary care clinics. The comparative success of the TB clinic may be the result of having staff that have been trained to focus on engagement of patients in LTBI treatment, whereas primary care visits often attempt to address multiple issues in a 15-min visit without dedicated staff to address LTBI treatment. Alternatively, the relative success of the public health TB clinic may be due in part to patient motivation to complete therapy because personal experience through close contact to active TB cases.

While a homeless incentive program was used in this study, we did not see a higher rate of completion in the homeless clinic compared to other sites. This is in line with recent findings that incentive programs may have short term effects on clinic attendance, but do not seem to reliably increase the number of people completing treatment for LTBI [27].

Our study has limitations. First, given that our study was retrospective and not randomized, the design limits the strength of the conclusions regarding the true equivalence of the regimens. Provider discretion should still guide the choices between rifampin only regimens versus INH-rifapentine, which may still be of greater utility in patient populations with historically low adherence, such as homeless patients and those actively using alcohol or other substances. Second, all participating clinics are located in one US city. Though we include a representative mix of foreign-born and homeless persons (see Table 1), our findings may not be generalizable to all populations in all settings. Further research is needed to clarify completion rates in other actual use settings such as other clinic settings, different cities, rural areas, and other states and countries. Third, there were more patients from the TB clinic than the other clinic sites, which may have limited our ability to detect differences in the smaller samples of the non TB clinic patients. Fourth, each clinic has unique patient populations and characteristics that limit the interpretation of comparisons among different clinics. These regimens have different roles in how they are employed in LTBI treatment programs depending on provider and patient preferences and needs. Fifth, treatment regimens also varied over time based on standard of care. Rifampin alone was used much less frequently in our medical center in 2009, as INH was the standard of care. Similarly, once our medical center made the switch to isonaizid plus rifapentine and rifampin only regimens, the number of people being treated with INH alone was quite small, and a simultaneous comparison of the three regimens using only 2013 data would have been underpowered to detect a difference. To adequately compare the three regimens, we pooled data from two different time points in order to have large enough sample sizes to detect a true difference. We did not set up our analysis to compare INH in 2009 to INH in 2013 because that was not the primary outcome of interest. Rather, the goal was to compare completion rates for INH, rifampin and INH plus rifapentine regimens in an “actual use” scenario (rather than research settings, where monitoring and compliance tend to be more rigorous than a “true” patient might actually adhere to). Sixth, our study was limited to adults and excluded individuals with HIV co-infection. Lastly, since our sample size was determined by the number of patients in our clinics we may have had insufficient power to detect associations. Population-based studies and randomized clinical trials are needed to clarify factors associated with non-completion of these regimens, and to assess side effects and adverse events.

## Conclusions

In conclusion, this outpatient-based comparison of four months of rifampin, three months of isoniazid and rifapentine, and nine months of isoniazid demonstrates comparable completion rates between four months of rifampin and three months of isoniazid and rifapentine while taking into consideration the monitoring frequency. Given the similar side effect profile but significant cost savings with four months of rifampin, we suggest that four months of rifampin be considered exchangeable with three months of isoniazid and rifapentine and nine months of isoniazid for treatment of latent tuberculosis infection.

## Abbreviations

DOT: Directly observed therapy
LTBI: Latent Tuberculosis infection
TB: Mycobacterium tuberculosis
